# mTOR Inhibition Induces Compensatory, Therapeutically Targetable MEK Activation in Renal Cell Carcinoma

**DOI:** 10.1371/journal.pone.0104413

**Published:** 2014-09-02

**Authors:** Sean T. Bailey, Bing Zhou, Jeffrey S. Damrauer, Bhavani Krishnan, Harper L. Wilson, Aleisha M. Smith, Mingqing Li, Jen Jen Yeh, William Y. Kim

**Affiliations:** 1 Lineberger Comprehensive Cancer Center, University of North Carolina at Chapel Hill, Chapel Hill, North Carolina, United States of America; 2 Department of Medicine, University of North Carolina at Chapel Hill, Chapel Hill, North Carolina, United States of America; 3 Department of Genetics, University of North Carolina at Chapel Hill, Chapel Hill, North Carolina, United States of America; 4 Department of Surgery, University of North Carolina at Chapel Hill, Chapel Hill, North Carolina, United States of America; 5 Department of Pharmacology, University of North Carolina at Chapel Hill, Chapel Hill, North Carolina, United States of America; Emory University, United States of America

## Abstract

Rapamycin derivatives allosterically targeting mTOR are currently FDA approved to treat advanced renal cell carcinoma (RCC), and catalytic inhibitors of mTOR/PI3K are now in clinical trials for treating various solid tumors. We sought to investigate the relative efficacy of allosteric versus catalytic mTOR inhibition, evaluate the crosstalk between the mTOR and MEK/ERK pathways, as well as the therapeutic potential of dual mTOR and MEK inhibition in RCC. Pharmacologic (rapamycin and BEZ235) and genetic manipulation of the mTOR pathway were evaluated by *in vitro* assays as monotherapy as well as in combination with MEK inhibition (GSK1120212). Catalytic mTOR inhibition with BEZ235 decreased proliferation and increased apoptosis better than allosteric mTOR inhibition with rapamycin. While mTOR inhibition upregulated MEK/ERK signaling, concurrent inhibition of both pathways had enhanced therapeutic efficacy. Finally, primary RCC tumors could be classified into subgroups [(I) MEK activated, (II) Dual MEK and mTOR activated, (III) Not activated, and (IV) mTOR activated] based on their relative activation of the PI3K/mTOR and MEK pathways. Patients with mTOR only activated tumors had the worst prognosis. In summary, dual targeting of the mTOR and MEK pathways in RCC can enhance therapeutic efficacy and primary RCC can be subclassified based on their relative levels of mTOR and MEK activation with potential therapeutic implications.

## Introduction

Recent statistics suggest that there are predicted to be roughly 65,000 new cases and 14,000 deaths in 2013 from renal cell carcinoma (RCC) [1], [2]. Clear cell renal cell carcinoma (ccRCC) is the most common histologic subtype of RCC and the vast majority of sporadic ccRCC have inactivation of the von Hippel-Lindau tumor suppressor protein (pVHL). Patients with VHL disease have inherited mutations of *VHL* and renal cyst and/or tumors develop when these individuals undergo somatic inactivation or loss of the remaining wild-type *VHL* allele [3], [4]. pVHL's most well understood function is to negatively regulate the hypoxia-inducible factor alpha (HIFα) family of transcription factors (HIF1α, HIF2α, HIF3α) in an oxygen dependent manner via its E3 ubiquitin ligase activity [5], [6]. Importantly, pVHL's tumor suppressor function is dependent upon the downregulation of HIFα subunits and in particular HIF2α [7]–[9].

Stabilization of HIFα, either as a consequence of hypoxia or pVHL inactivation leads to transcriptional activation of numerous genes associated with adaptation to a hypoxic environment as well as an unfavorable tumor microenvironment [2], [5], [10]. The development of FDA approved therapies for combating ccRCC has been heavily influenced by an understanding of the molecular underpinnings of VHL disease. Specifically, small-molecule tyrosine kinase inhibitors (e.g. sunitinib and pazopanib) have been developed to inhibit vascular endothelial growth factor receptor (VEGFR) and platelet derived growth factor receptor (PDGFR) [3], [10]. Additionally, temsirolimus and everolimus, derivatives of rapamycin, are approved to treat advanced RCC [5]. While significant tumor responses are seen in the setting of VEGFR inhibition they are much less common upon mTOR inhibition suggesting potential compensatory survival and proliferative mechanisms that can be co-targeted [11], [12].

Rapamycin and its derivatives are allosteric inhibitors of the serine/threonine kinase, mechanistic target of rapamycin (mTOR), that require rapamycin's association with cytosolic protein, FKBP12 [5], [13]. mTOR integrates extracellular growth signals with cellular responses such as proliferation, autophagy, metabolism, cell growth and survival [14]. The mTOR protein kinase interacts with several proteins to form two distinct complexes, mTORC1 and mTORC2. Both mTORC1 and mTORC2 are composed of the common subunits: DEP domain containing mTOR-interacting protein (DEPTOR), mammalian lethal with sec-13 protein 8 (mLST8), and tti1/tel2 complex. However, they differ in composition by several additional proteins. Regulatory-associated protein of mammalian target of rapamycin (Raptor) and proline-rich AKT substrate 40 KDa (PRAS40) are distinct to the mTORC1 signaling complex while rapamycin-insensitive companion of mTOR (Rictor), mammalian stress-activated map kinase-interacting protein1 (mSin1), and protein observed with Rictor 1 and 2 (protor1/2) are associated with mTORC2 [15]. Notably, the mTORC2 complex is thought to be relatively insensitive to rapamycin [16]. Furthermore, treatment with rapamycin and it's derivatives causes a release of negative feedback on the PI3K/AKT signaling pathway [17], [18]. Therefore, the inability of rapamycin to inhibit all signaling nodes of mTOR has warranted efforts to develop catalytic mTOR inhibitors capable of perturbing mTOR's kinase activity and therefore blocking both mTORC1 and mTORC2 complexes [19].

However, recent reports have demonstrated that inhibitors of mTOR are capable of increasing MEK/ERK activation and its associated proliferation and survival signaling in cancer cells [20]–[26]. Interestingly, several groups have observed that catalytic mTOR inhibition increases compensatory MEK/ERK signaling greater than allosteric mTOR inhibition [23], [27]. This particular observation has resulted in pre-clinical and clinical studies utilizing mTOR inhibition in combination with MEK inhibition for treating several cancer types [26], [28]–[30]

Here, we investigate, through both a pharmacologic and genetic approach, the compensatory proliferation and survival pathways observed in the context of allosteric and catalytic mTOR inhibition. The studies conducted here support that catalytic mTOR inhibition may be better than allosteric inhibition at restraining cellular proliferation and increasing apoptosis. However, we also observe that catalytic mTOR inhibition is more robust at initiating compensatory MEK/ERK signaling in RCC. We address these compensatory cross-talk pathways through pharmacologic inhibition and demonstrate that the selected combinatorial approaches reveal an enhanced effect at attenuating cellular proliferation and augmenting the apoptotic response in RCC cells.

## Results

### Novel renal cell carcinoma cell lines lack VHL and overexpress HIF

**Figure 1:**
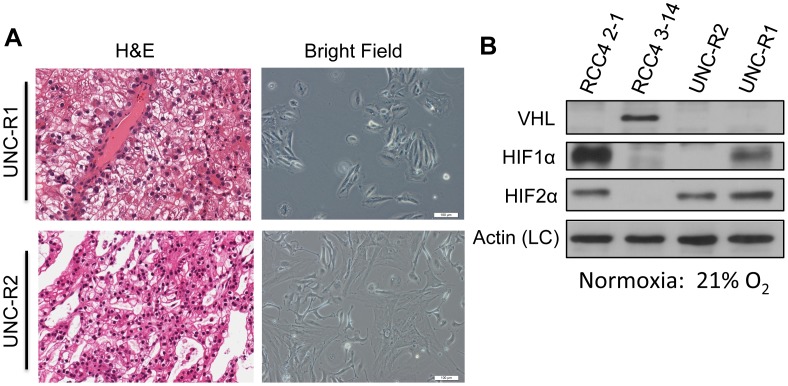
Novel renal cell carcinoma cell lines lack VHL and overexpress HIF. (**A**) Photomicrographs of H&E stains (left panels) and bright field images (right panels) of UNC-R1 and UNC-R2 PDX derived cell lines. (**B**) Whole cell extracts from UNC-R1 and UNC-R2s were immunoblotted with the indicated antibodies. RCC4 2-1 (VHL null) and RCC4 3–14 (VHL positive) were included as controls.

In order to aid our studies, we generated two novel ccRCC cell lines (hereafter called UNC-R1 and UNC-R2) from primary patient-derived xenografts (PDX). H&E staining of a portion of the PDX tumor demonstrated clear cell histology (Figure 1A). Cell morphology of the cell lines remained consistent over time. To characterize these novel cell lines, VHL, HIF1α, and HIF2α expression were determined by western blot (Figure 1B). RCC4 2-1 (VHL null) and RCC4 3–14 (VHL wt) were used as controls to validate current findings. Both the UNC-R1 and the UNC-R2 cell lines lacked appreciable expression of VHL. While both cells lines expressed HIF2α, only UNC-R1 expressed HIF1α (Figure 1B), suggesting that UNC-R2 cells have lost HIF1α expression as is seen in a proportion of ccRCC cell lines and primary tumors and consistent with the notion that HIF1α is potentially a tumor suppressor gene [31].

### Catalytic mTOR inhibitors block mTORC1 signaling more fully than allosteric mTOR inhibition

**Figure 2:**
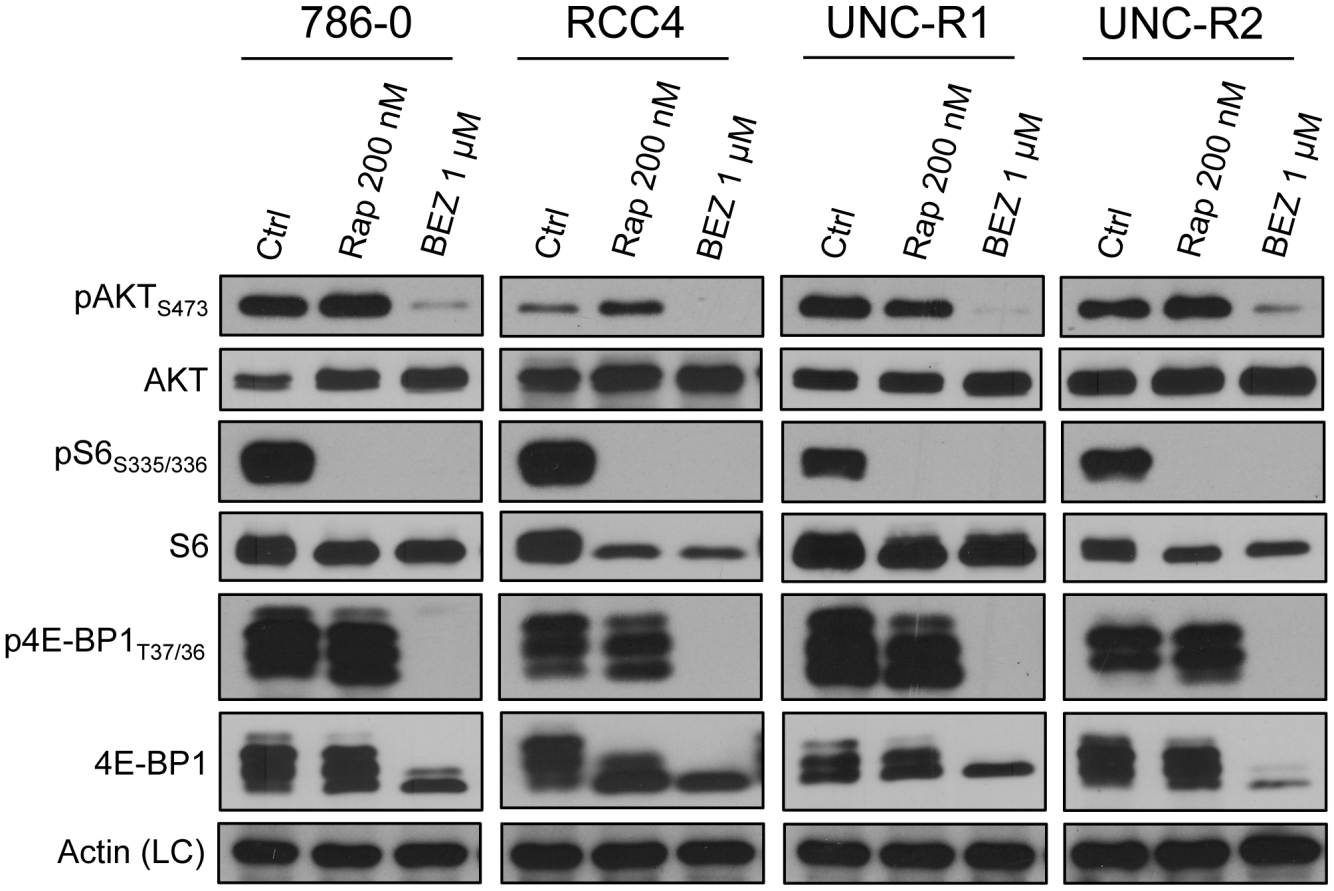
Catalytic mTOR inhibitors block mTORC1 signaling more fully than allosteric mTOR inhibition. The indicated cell lines were treated with the allosteric and catalytic mTOR inhibitors (rapamycin and BEZ235 respectively) at the indicated concentrations for 24 hrs. Whole cell extracts were then immunoblotted with the indicated antibodies.

Previous studies have demonstrated that the dual catalytic PI3K/mTOR inhibitor, BEZ235, inhibits mTORC1 signaling better than allosteric mTOR inhibition with rapamycin or other rapalogs [32]–[36]. We wished to see whether these results could be replicated in our hands. Dose titrations of rapamycin and BEZ235 in a panel of human RCC cell lines showed that 200 nM and 1 uM respectively were required to inhibit mTORC1 and/or mTORC2 signaling (Figure S1A and S1B). These doses of rapamycin and BEZ235 were therefore used to treat a panel of RCC cell lines. As expected, while both compounds inhibited the phosphorylation of S6, only BEZ235 inhibited phosphorylation of 4EBP1 and AKT Ser 473 (Figure 2). Moreover, as previously described, allosteric mTOR inhibition with rapamycin resulted in increased pAKT_S473_ expression in 786-0, RCC4, and UNC-R2 cells (Figure 2) presumably as a result of release of S6K and IRS1 dependent negative feedback inhibition of PI3K/AKT signaling [17].

### Catalytic mTOR inhibition is superior to allosteric mTOR inhibition at attenuating cellular proliferation and inducing apoptosis

**Figure 3:**
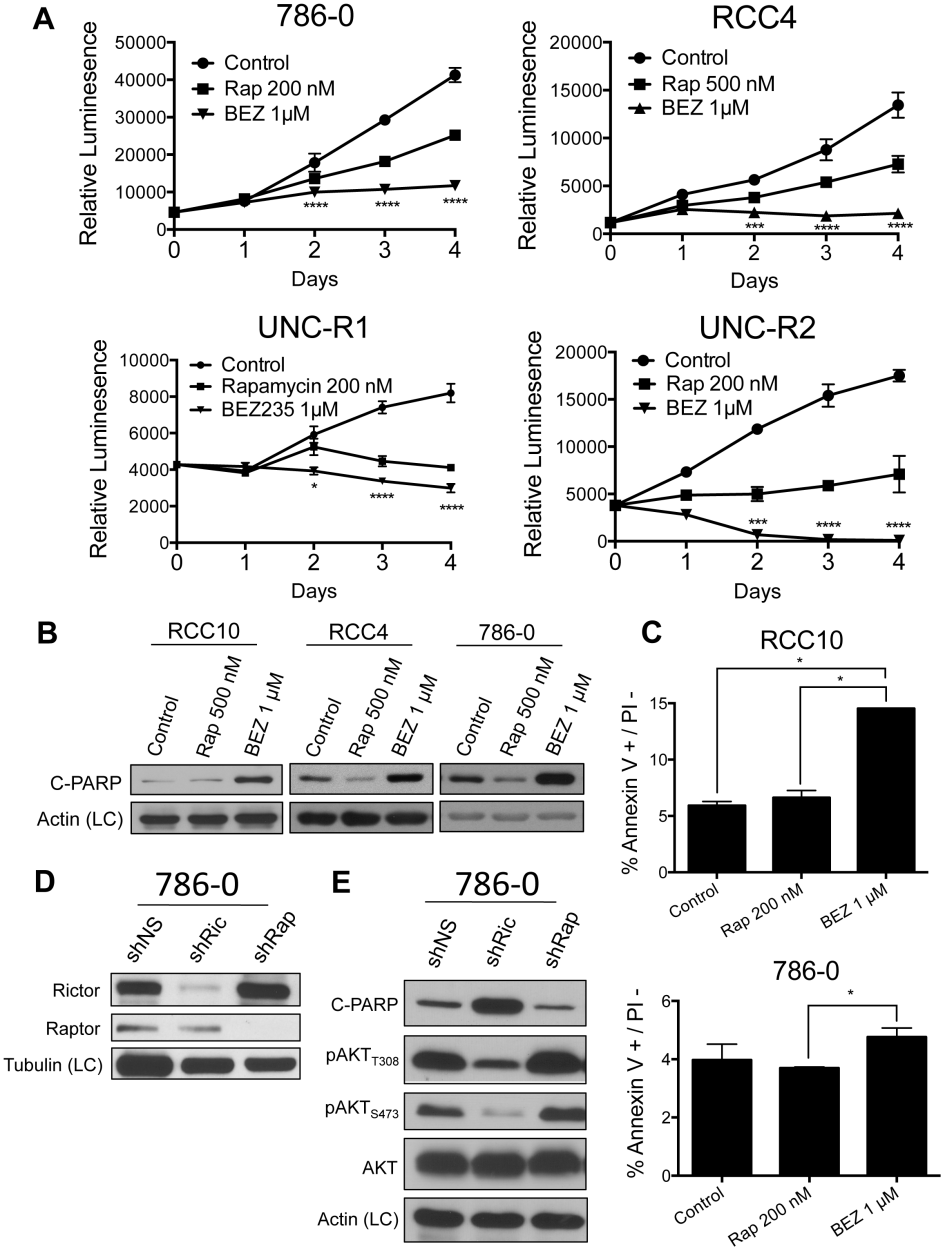
Catalytic mTOR inhibition attenuates proliferation and induces apoptosis better than allosteric mTOR inhibition. (**A**) The indicated cell lines were assessed for viability on the indicated days using CellTiter-Glo. Statistical significance was determined by comparing rapamycin and BEZ235 treated groups. (**B**) The indicated cell lines were treated with rapamycin and BEZ235 for 48 hours and immunoblotted with the indicated antibodies. (**C**) The indicated cell lines were treated with rapamycin and BEZ235 for 48 hours and assessed for apoptosis by flow cytometry analysis of the Annexin V+/PI – fraction. (**D**) 786-0 cells were stably infected with shRNAs targeting Raptor (mTORC1) or Rictor (mTORC2) and confirmed for knock-down by western blot. (**E**) Whole cell extracts from 786-0 shNS, shRaptor, and shRictor cells were immunoblotted with the indicated antibodies.

Previous groups have demonstrated that BEZ235 is better than rapamycin at decreasing cellular proliferation in RCC [33]. We utilized CellTiter-Glo to measure alterations in cellular viability over the course of 4 days. Consistent with previous results, our data show that BEZ235 inhibits cellular proliferation better than rapamycin (Figure 3A and Figure S2). Interestingly, both primary cell lines (UNC-R1 and UNC-R2), but especially UNC-R2, seemed exquisitely sensitive to BEZ235 as evidenced by significantly fewer cells present at day 4 than to day 0 (Figure 3A).

While they prolong overall survival, allosteric mTOR inhibitors such as everolimus and temsirolimus have displayed little cytotoxic effects in patients (i.e. they lead to few objective responses) [12]. Catalytic mTOR inhibitors have shown increased efficacy in generating an apoptotic response in preclinical studies, likely as a result of decreasing AKT mediated survival signals [32], [34]. Treatment of RCC4, 786-0, and RCC10 cells with BEZ235 resulted in increased apoptosis as evidenced by the increased expression of the apoptotic marker, cleaved PARP (poly ADP ribose polymerase) (Figure 3B). Moreover, BEZ235 also increased expression of another apoptotic marker, cleaved-caspase 3, in RCC4 and RCC10 cells (Figure S3). Interestingly, 786-0 and RCC4 cells showed a decrease in cleaved-PARP expression when treated with rapamycin (Figure 3B) likely as a consequence of the increased survival signaling from AKT (Figure 2). Additionally, assessment of apoptosis by flow cytometry (Annexin V+/PI- fraction) also showed that RCC cells treated with BEZ235 had increased apoptosis respective to rapamycin treated cells (Figure 3C). Therefore, catalytic mTOR inhibition is superior to allosteric mTOR inhibition at attenuating cellular proliferation and inducing apoptosis.

### mTORC2 activity negatively regulates the apoptotic response through phosphorylation of AKT

We wanted to determine whether the enhanced apoptosis seen with BEZ235 treatment (relative to rapamycin) was due to its ability to inhibit mTORC2 and subsequent downregulation of AKT dependent survival signaling. Since there are no pharmacologic inhibitors capable of specifically inhibiting mTORC2, we silenced Rictor expression, which is required for mTORC2 activity (Figure 3D). Knock-down of Rictor significantly decreased expression of pAKT_S473_ and pAKT_T308_ as well as increased cleaved-PARP (Figure 3E). In contrast, knock-down of Raptor, which is required for mTORC1 activity, appeared to slightly decrease cleaved-PARP expression while mildly increasing pAKT_S473_ or pAKT_T308_ expression. These results support the notion that the increased apoptosis seen with BEZ235 relative to rapamycin treatment are a result of BEZ235's inhibition of mTORC2 activity.

### mTOR inhibition induces compensatory activation of MEK/ERK signaling

**Figure 4:**
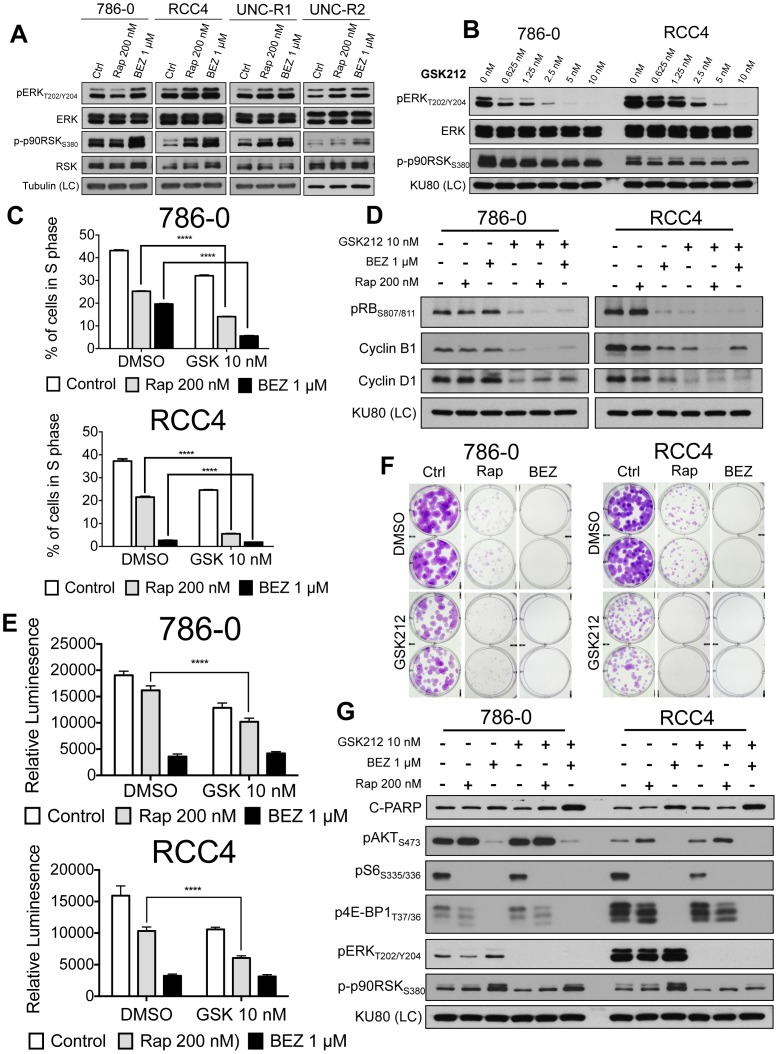
Combined mTOR and MEK inhibition attenuates cellular proliferation and increases the apoptotic response. (**A**) The indicated cells were treated for 24 hrs. with rapamycin or BEZ235 and immunoblotted with the indicated antibodies. (**B**) 786-0 and RCC4 cells were treated increasing doses of GSK212 for 24 hrs. and immunoblotted with the indicated antibodies. (**C**) 786-0 and RCC4 cells were treated for 24 hrs with rapamycin and BEZ235 in the presence of Edu. Edu incorporation was assessed by flow cytometry. (**D**) 786-0 and RCC4 cells were treated with the indicated compounds for 24 hrs. Whole cell extracts were immunoblotted for the cell cycle related proteins indicated. (**E**) 786-0 and RCC4 cells were treated with indicated drugs and assessed for viability on day 4 using CellTiter-Glo 4. (**F**) 786-0 and RCC4 cells were plated, allowed to attach, and treated with the indicated drug(s). Photographs of wells containing 786-0 (day 11) and RCC4 (day 17) cells fixed with 4% PFA and stained with 0.1% crystal violet. (**G**) 786-0 and RCC4 cells were treated with the indicated compounds for 24 hrs. Whole cell extracts were immunoblotted with the indicated antibodies.

Recent reports have demonstrated cross-talk between the mTOR and MEK/ERK signaling pathways [37]. To see whether this interplay was present in the context of RCC we examined the response of ERK and a canonical ERK substrate, p90RSK, to rapamycin or BEZ235. Both pERK and p-p90RSK were induced by allosteric and catalytic mTOR inhibition (Figure 4A). While there was a sense that BEZ235 treatment resulted in a slightly larger increase in p-p90RSK in a subset of the cell lines, this was not accompanied by the same amount of induction of pERK. This could reflect enhanced ERK activity that is not appreciable by pERK western blotting or mTOR inhibition induced p-p90RSK that is ERK independent. However, ERK is the only described kinase to phosphorylate p90RSK on the S380 site [38]. Overall, these results suggest that mTOR inhibition of RCC cells upregulates MEK/ERK signaling and that catalytic mTOR inhibition may do so in a more robust manner than allosteric inhibition.

### Combination of mTOR and MEK inhibition attenuates cellular proliferation and increases the apoptotic response

The observation of increased MEK/ERK signaling in the context of mTOR inhibition led us to hypothesize that attenuation of this compensatory signal may decrease cellular proliferation and induce apoptosis. We saw that 10 nM of the MEK inhibitor GSK1120212 (hereafter called GSK212) was sufficient to fully inhibit MEK activity as assessed by pERK _T202/Y204_ in several RCC cell lines (Figure 4B). Treatment of RCC cell lines with rapamycin or BEZ235 led to a decrease in the percentage of cells in S phase as determined by Edu incorporation (Figure 4C). The combination of MEK inhibition with mTOR inhibition led to a potent reduction in S phase fraction, particularly when GSK212 was combined with BEZ235. As expected, MEK inhibition led to hypophosphorylation of Rb as well as downregulation of cyclin B1 and cyclin D1 consistent with increased cell cycle arrest. However, the addition of mTOR inhibition did not further change levels of these proteins (Figure 4D).

Despite the fact that the combination of BEZ235 and GSK212 potently inhibited cell cycle progression, there did not appear to be an additive effect on proliferation or colony formation (Figure 4E and 4F). We hypothesized that this lack of additivity was secondary to the high level of inhibition of proliferation and colony formation by 1 µM BEZ235 alone. Therefore, we determined the IC50 for BEZ235 in several RCC cell lines (Figure S4A), confirmed that the determined IC50 was capable of inducing activation of MEK/ERK signaling (Figure S4B), and examined its effects on proliferation on colony formation. The combination of 2 nM of BEZ235 with GSK212 resulted in significant decreases in proliferation (Figure S5A) and colony formation (Figure S5B) over either single agent alone. Furthermore, the combination of mTOR inhibition with MEK inhibition augmented the apoptotic response as evidence of increased C-PARP expression in 786-0 and RCC4 cells treated with the combination (Figure 4G). Together, these data support the notion that combined mTOR and MEK inhibition might be an effective therapy in RCC.

### Subclasses of RCC can be defined by MEK and mTOR pathway activation

**Figure 5:**
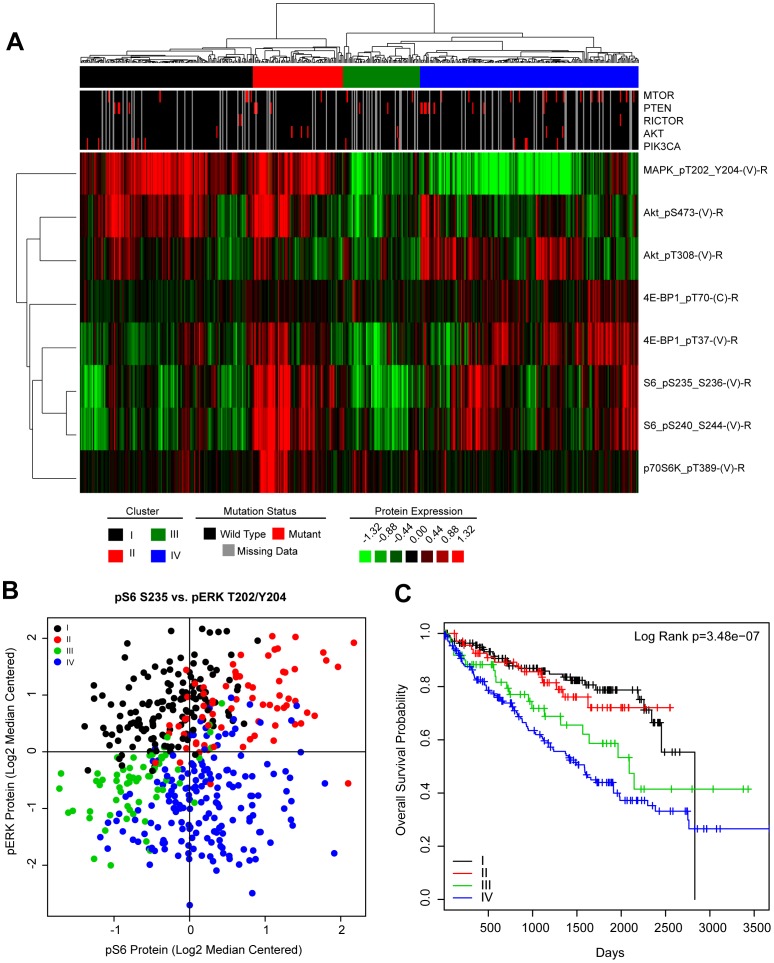
Subclasses of RCC can be defined by MEK and mTOR pathway activation. (**A**) TCGA KIRC RPPA data was log2 transformed, median centered. Tumors were then hierarchically clustered and the indicated subgroups were determined based on expression patterns of the indicated phosphoproteins. Mutational data for mTOR pathway related genes were annotated in the upper tracks. (**B**) Scatter plot of TCGA KIRC tumors based on expression of pS6 and pERK. Each dot indicates a tumor. The MEK-PI3K/mTOR subclasses defined in (**A**) are indicated by color. (**C**) Patients harboring tumors within each MEK-PI3K/mTOR subclass were evaluated for differences in overall survival by the Log Rank test and shown as a Kaplan-Meier plot of overall survival.

To assess the potential relevance of MEK and/or mTOR inhibition in ccRCC we examined reverse phase protein array data (RPPA) from the TCGA clear cell kidney cancer project (KIRC) to determine the relative activation state of these pathways in human RCC [39]. Reverse phase protein arrays are a highly validated technique allowing the assessment of protein expression across hundreds of proteins simultaneously and because of the multiplatform nature of the TCGA allows for correlations to other genomic aspects of a tumor. To this end, TCGA KIRC tumors were hierarchically clustered using log2 transformed, median centered, RPPA expression data of canonical phosphoproteins that represent activation of the MEK (pERK_T202/Y204_), PI3K (pAkt_T308_), mTORC1 (p4E-BP1_T70_, p4E-BP1_T37_, pS6_S235/236_, pS6_S240/244_, p70S6K_T389_) and mTORC2 (pAkt_S473_) pathways (Figure 5A). There were 4 well-defined clusters of tumors that appeared to represent differential patterns of MEK and mTOR activation: 1) MEK activation [I: black]. 2) dual MEK and mTOR activation [II: red]. 3) no activation [III: green]. and 4) mTOR activation [IV: blue]. These subgroups could be also visualized using a plot that graphed the relative expression of the canonical markers of MEK activation (pERK_T202/Y204_) and mTORC1 activation (pS6_S235_) (Figure 5B).

Finally, we wanted to see if our MEK/mTOR subgroups held prognostic value and thus assessed their patterns of overall survival. We found that patients with mTOR activated tumors (IV: blue) had the worse overall survival while patients with high MEK activation, regardless of mTOR status, had the best survival (I: black and II: red) (Figure 5C). Patients with RCC tumors without activation (III: green) had an intermediate overall survival. Therefore, subclasses of RCC tumors can be identified based on their relative activation of the MEK and mTOR pathways and the subclasses correlate with prognosis.

## Discussion

Our studies investigate the relative efficacy of allosteric versus catalytic mTOR inhibition in RCC through both pharmacologic and genetic approaches. We show that as monotherapy, catalytic mTOR inhibition is better at decreasing cellular proliferation and inducing apoptosis than allosteric mTOR inhibition consistent with previous studies in RCC [33]. However, despite these potentially therapeutically beneficial characteristics, we show that catalytic mTOR inhibition also induces a more robust induction of compensatory MEK/ERK signaling. Nonetheless, the compensatory upregulation in MEK/ERK signaling can be targeted with small molecule kinase inhibition, resulting in enhanced therapeutic efficacy. Finally, we demonstrate that primary RCC tumors can be classified based on their relative activation of the MEK and mTOR pathways and that these different MEK/mTOR subtypes are associated with differences in overall survival.

Dual inhibition of the MEK and PI3K/mTOR pathways has shown preclinical promise as a therapeutic strategy in a variety of tumors [24]–[26], [28], [40]–[42] and has entered into phase 1 trials in humans [29]. Inhibition of the MEK/ERK and PI3K/mTOR pathways is a rational strategy based on the extensive crosstalk between the two pathways and the well documented compensatory signaling that occurs in the face of MEK or mTOR inhibition [37]. Nonetheless, neither dual inhibition nor the compensatory cross-talk between the MEK and PI3K/mTOR pathways has been explored specifically in the context of RCC where it is highly clinically relevant given the approval of the allosteric mTOR inhibitors everolimus and temsirolimus for patients with advanced disease [12]. Therefore, our studies are the first to investigate this crosstalk and its potential clinical relevance in RCC.

Our studies showed that mTOR inhibition in RCC cell lines resulted in increased MAPK signaling in the context of both allosteric and catalytic mTOR inhibition (Figure 4A). Moreover, we noted that catalytic mTOR inhibition enhanced ERK phosphorylation, as well as phosphorylation of the ERK substrate, p90RSK more than robustly than allosteric mTOR inhibition (Figure 4A and G). Precisely how mTOR inhibition in RCC results in increased MEK/ERK signaling remains to be determined. Further investigation into this is warranted but overall our results are consistent with the notion that kinase inhibition results in upregulation of compensatory pathways and kinome reprogramming [43].

Examination of the RPPA data from the TCGA KIRC project allowed us to assess the possibility that RCC could be divided into subclasses based on the relative activation of the MEK and mTOR pathways as well as evaluate their potential therapeutic significance [39]. We have named these groups, MEK activated, mTOR activated, dual MEK and mTOR activated, and not activated. We propose that rational targeted therapy for the MEK/mTOR subgroups might include: MEK activated – MEK inhibitor, mTOR activated – allosteric or catalytic mTOR inhibitor, dual MEK and mTOR activated – combination MEK and mTOR inhibitor, and not activated – VEGFR tyrosine kinase inhibitor.

Patients with mTOR activated tumors had the worse overall survival relative to the other subgroups and are also the only subgroup that would be predicted to benefit from single agent, allosteric mTOR inhibition. Intriguingly, temsirolimus has been shown in a phase III randomized trial to prolong the overall survival of patients with “poor prognosis” as defined by the MSKCC criteria [12], [44]. While we cannot be sure that our mTOR activated group corresponds to the “poor prognosis” patients defined by the MSKCC criteria, if they do correlate, our data provides a biological explanation for this interesting clinical observation.

In summary, our studies demonstrate that catalytic mTOR inhibition is more effective than allosteric, but that catalytic mTOR inhibition appears to more robustly induce alternative compensatory pathways (i.e. MEK/ERK). Nonetheless, compensatory upregulation of MEK/ERK signaling can be co-targeted with enhanced therapeutic effectiveness. Furthermore, we describe distinct subclasses of RCC that can be defined by the activation of the MEK and mTOR pathways, have clinically distinct prognosis, and would be predicted to have differential responses to MEK and mTOR kinase inhibition. In aggregate, our data suggests that catalytic mTOR inhibition should be investigated in RCC and that the compensatory upregulation of MEK/ERK signaling may actually be a potential synthetic vulnerability in RCC.

## Materials and Methods

### Patient-derived xenograft cell isolation

Xenografts were excised and washed in a solution of Pen-Strep, 1XPBS solution (1∶1). In sterile conditions, xenografts were then cut into small 2×2 mm fragments and dissociated in gentleMACS C-Tube (Miltenyi Biotec) using the gentleMACs Dissociator (program: m_imp Tumor_02) in 5 mL of complete DMEM. Then 100 µL of collagenase D/dispase II (Roche: 40 mg/mL) was added to the tumor fragments and continuously inverted for 30 min at 37°C. Fragments were then subjected to another round of dissociation using the gentleMACS Dissociator (program: m_imp Tumor_03). 5 mL of protein extraction buffer (PEB: buffer 0.5% FBS, 2 mM EDTA in PBS) was added to the dissociated fragments and resuspended by pipetting. The cell suspension was transferred to a 50 mL conical tube through a 40 µm nylon mesh sterile cell strainer (Fisher). An additional 20 mL of PEB buffer was added to the cell suspension and it was then centrifuged at 300 g's for 5 min. Supernatant was removed and cell pellet was resuspended in 6 mL of complete DMEM and placed in a 6 cm sterile cell culture plate. De-identified tumor tissue was obtained from the University of North Carolina Institutional Review Board (IRB) approved tissue procurement facility after University of North Carolina IRB approval. The animal work was approved by the University of North Carolina Institutional Animal Care and Use Committee.

### Cell lines and culture conditions

RCC10, 786-0, RCC4, UNC-R1, UNC-R2 were cultured in complete DMEM (CORNING-Cellgro #10-013-CV) supplemented with 10%FBS, 1× Penn/Strep at 37°C, 5% CO_2_, 21% O_2_. 786-0 cells were obtained from ATCC. RCC4 cells were obtained from Dr. Kimryn Rathmell [45] and RCC10 cells were obtained from Dr. Michael Ohh [46]. RCC tumor tissue from de-identified patients were obtained from the University of North Carolina Institutional Review Board (IRB) approved tissue procurement facility after IRB approval. UNC-R1 and UNC-R2 cell lines were generated as above from the renal patient derived xenografts. BEZ235 (Center for Integrative Chemical Biology & Drug Discovery, UNC Eshelman School of Pharmacy), GSK1120212 (GlaxoSmithKline), Rapamycin (LC Laboratories) were dissolved in DMSO.

### Immunoblot conditions

Cells were lysed in RIPA buffer complemented with Set I and Set II phosphatase inhibitors at 1× (Calbiochem), and protease inhibitors at 1× (Roche). Whole cell lysate concentration was determined with Bio-Rad Protein Assay Dye Reagent Concentrate (Bio-Rad). Proteins were resolved on SDS-PAGE gels and electrotransferred to nitrocellulose membranes, 0.2 µm (Bio-Rad). Primary antibodies pS6_S235/236_, S6, pAKT_S473_, AKT, p4E-BP1_T37/46_, 4E-BP1, Cleaved PARP, pAkt_T308_, p62, Rictor, Raptor, HIF-1α, HIF-2α, pERK1/2_T202/Y204_ (mouse), ERK, p-p90RSK_S380_, RSK1/2/3, pBAD_S112_, pBAD_S136_, pEGFR_Y1068_, cleaved-caspase3 were from Cell Signaling Technologies. VHL (Santa Cruz #FL-181). mTOR primary antibody was from Millipore. Primary antibody dilutions were to manufactures' specifications (See Table S1). Tubulin (Sigma #T5168), KU-80 (GeneTex #GTX70485) and Actin-HRP (Santa Cruz #C-11) primary antibodies served as loading controls (LC) where noted. Secondary anti-Rabbit and ant-mouse antibodies were from (Fisher) and diluted in 5% milk, 1× TBS-T solution. ECL Western Blotting Detection reagents (GE Healthcare) were used for developing blots onto autoradiography film. For difficult to detect proteins SuperSignal West Femto Maximum Sensitivity Substrate (Thermo Scientific) was used in combination with ECL.

### Cell viability assay

To determine cell viability in the context of the various culture conditions we used a CellTiter-Glo Luminescent Cell Viability Assay (Promega) per manufacture's protocol. Cells were counted and plated in quadruplicate in a 96 well opaque side/clear bottom cell culture plates (Corning) in culture medium containing the noted concentration. Luminescence measurements were captured using a Biotek Synergy 2 plate reader. 2-way ANOVA analysis was used to determine statistical significance.

### Cell cycle analysis by flow cytometry

Cells were plated in triplicate and treated for 24 hrs with indicated drug. Cell cycle analysis was performed by EdU incorporation using Click-iT EdU Alexa Fluor 647 flow assay kit (Invitrogen, catalog number C-10424) according to the manufacturer's instructions. After treatment cells were exposed to 10 µM EdU for 2 h. Cells were then dissociated with 0.05% Trypsin/EDTA and fixed immediately, with 4% PFA, for incorporated EdU detection. Total DNA content was stained with propidium iodide (PI) at 10 µg/ml after RNase A treatment. Flow cytometry was performed on a CyAnTM ADP flow cytometer (Dako, Glostrup, Denmark) and data analysis was performed using FlowJo software (Tree Star, Inc.). Statistical significance was measured by Student's T-Test.

### Apoptosis analysis by flow cytometry

Cells were plated in duplicate and treated with the indicated drug for 48 hrs. Percentage of apoptotic cells were determined by staining with Annexin V Alexa Fluor 488 & PI (Dead Cell Apoptosis Kit, Invitrogen, cat# V13241) according to the manufacturer's instructions. Flow was performed on a Dako CyAn ADP and data were analyzed using FlowJo software. Statistical significance was measured by Student's T-Test.

### RNAi experiments

pLKO.1 shRNA plasmids were obtained form the UNC Viral Vector Core, packaged and infected per manufacture's protocols. Addgene catalogue numbers: shNS (#1864), shRictor (#1853), shRaptor (#1857), shmTOR (#1853). Cells were incubated with viral media over-night, and replaced with fresh complete media. Selection with 1 µg/mL puromycin was started 48 hrs later.

### Colony formation assay

RCC cells were plated at low-density in a 6 well plate (786-0: 50 cell/well and RCC4: 100 cells/well). Cells were allowed to attach and treated with indicated drug(s). Treatment conditions were changed every 72 hrs. Cells were fixed with 4% PFA and stained with crystal violet.

### TCGA data analysis

TCGA KIRC RPPA protein data was log2 transformed and median centered. Tumors samples (n = 454) and proteins relating to ERK and mTOR signaling were hierarchically clustered by centroid linkage using Cluster 3.0 and protein clusters were determined based on tumors sharing a common node. Mutations in mTOR related genes were annotated and superimposed as tracks above the heatmap for visualization. Scatter and Kaplan-Meier plots were generated in R (http://cran.r-project.org). Survival differences were determined by log-rank test.

## Supporting Information

Figure S1 **Dose titrations of rapamycin and BEZ235.** The indicated cell lines were treated with increasing doses of rapamycin (**A**) or BEZ235 (**B**) for 24 hours. Whole cell extracts were immunoblotted with the indicated antibodies to evaluate changes in mTORC1 and mTORC2 signaling.
(TIF)

Figure S2 **Proliferation curve of RCC10 cells.** RCC10 cells were treated over the course of 4 days with the indicated drugs and assessed for viability using CellTiter-Glo.
(TIF)

Figure S3 **Apoptosis in response to rapamycin or BEZ235 treatment.** RCC10 and RCC4 cells treated with rapamycin or BEZ235 for 24 hrs and analyzed by western blot for apoptotic marker cleaved-caspase 3.
(TIF)

Figure S4 **Dose titrations of BEZ235.** (**A**) 786-0 and RCC4 cells were plated and treated with a dose titration of BEZ235 and IC50 value determined using CellTiter-Glo cell viability reagent. (**B**) 786-0 and RCC4 cells were treated with 2 nM BEZ235 over a 24 hr. time course and immunobloted for protein expression of mTORC1, mTORC2, and MEK/ERK signaling proteins.
(TIF)

Figure S5 **Combinatorial effects of mTOR and MEK inhibition.** (**A**) 786-0 and RCC4 cells were treated with indicated drugs and assessed for viability on day 4 using CellTiter-Glo. Statistical significance was determined by comparing rapamycin and BEZ235 treated groups (**B**) 786-0 and RCC4 cells were plated, allowed to attach, and treated with 200 nM rapamycin, 2 nM BEZ235, 10 nM GSK212. Photographs of wells containing 786-0 and RCC4 cells fixed with 4% PFA and stained with 0.1% crystal violet.
(TIF)

Table S1 **List of antibodies used including company and catalogue number.**
(DOCX)
