# Supplementary figures and images for: mTOR Inhibition Induces Compensatory, Therapeutically Targetable MEK Activation in Renal Cell Carcinoma
Source: PLoS One. 2014 Sep 2;9(9):e104413. doi: 10.1371/journal.pone.0104413 (PMC4152178; doi:10.1371/journal.pone.0104413)

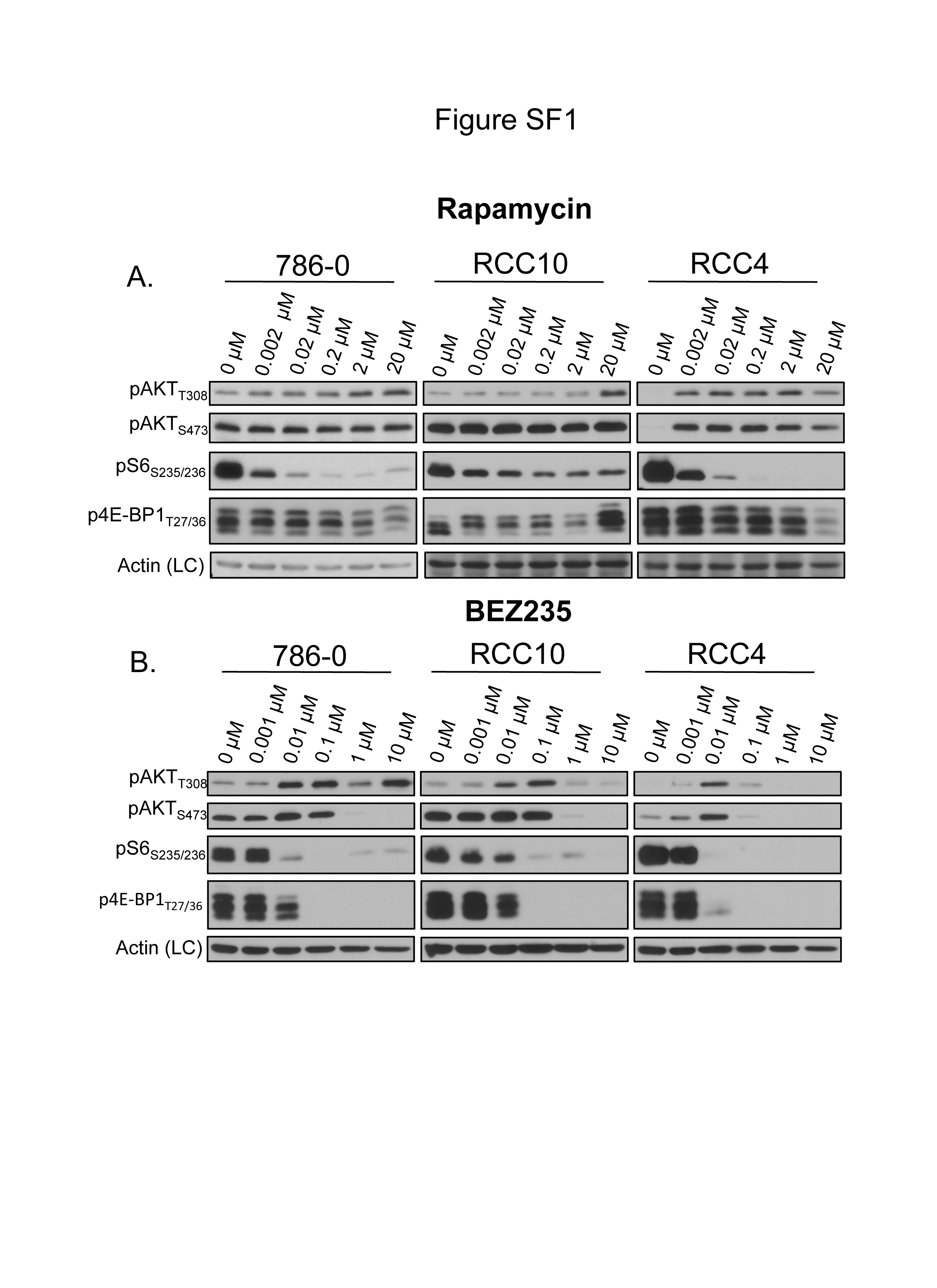

Supplement: Figure S1 — Dose titrations of rapamycin and BEZ235. The indicated cell lines were treated with increasing doses of rapamycin (A) or BEZ235 (B) for 24 hours. Whole cell extracts were immunoblotted with the indicated antibodies to evaluate changes in mTORC1 and mTORC2 signaling. (TIF) [file pone.0104413.s001.tif]

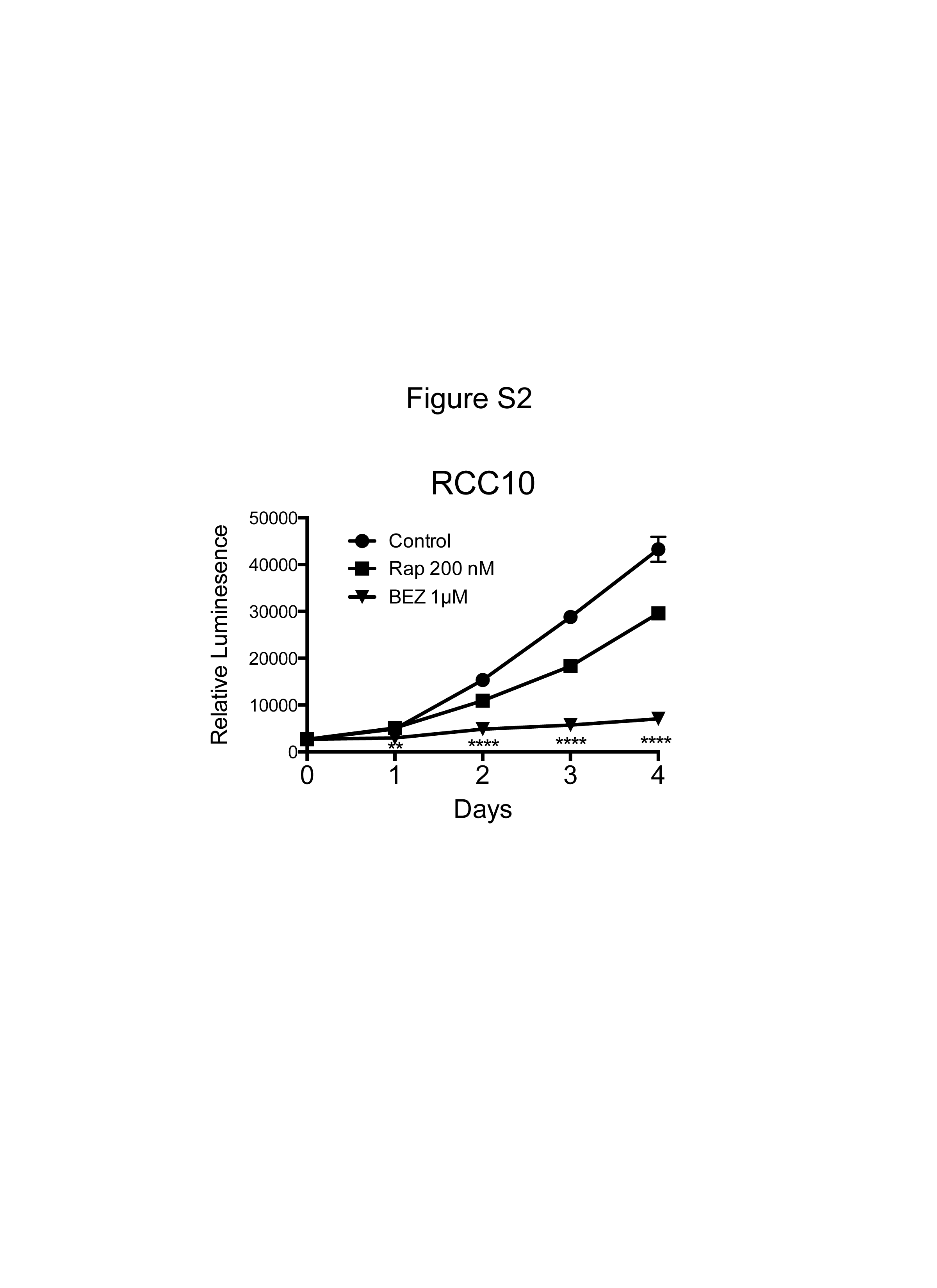

Supplement: Figure S2 — Proliferation curve of RCC10 cells. RCC10 cells were treated over the course of 4 days with the indicated drugs and assessed for viability using CellTiter-Glo. (TIF) [file pone.0104413.s002.tif]

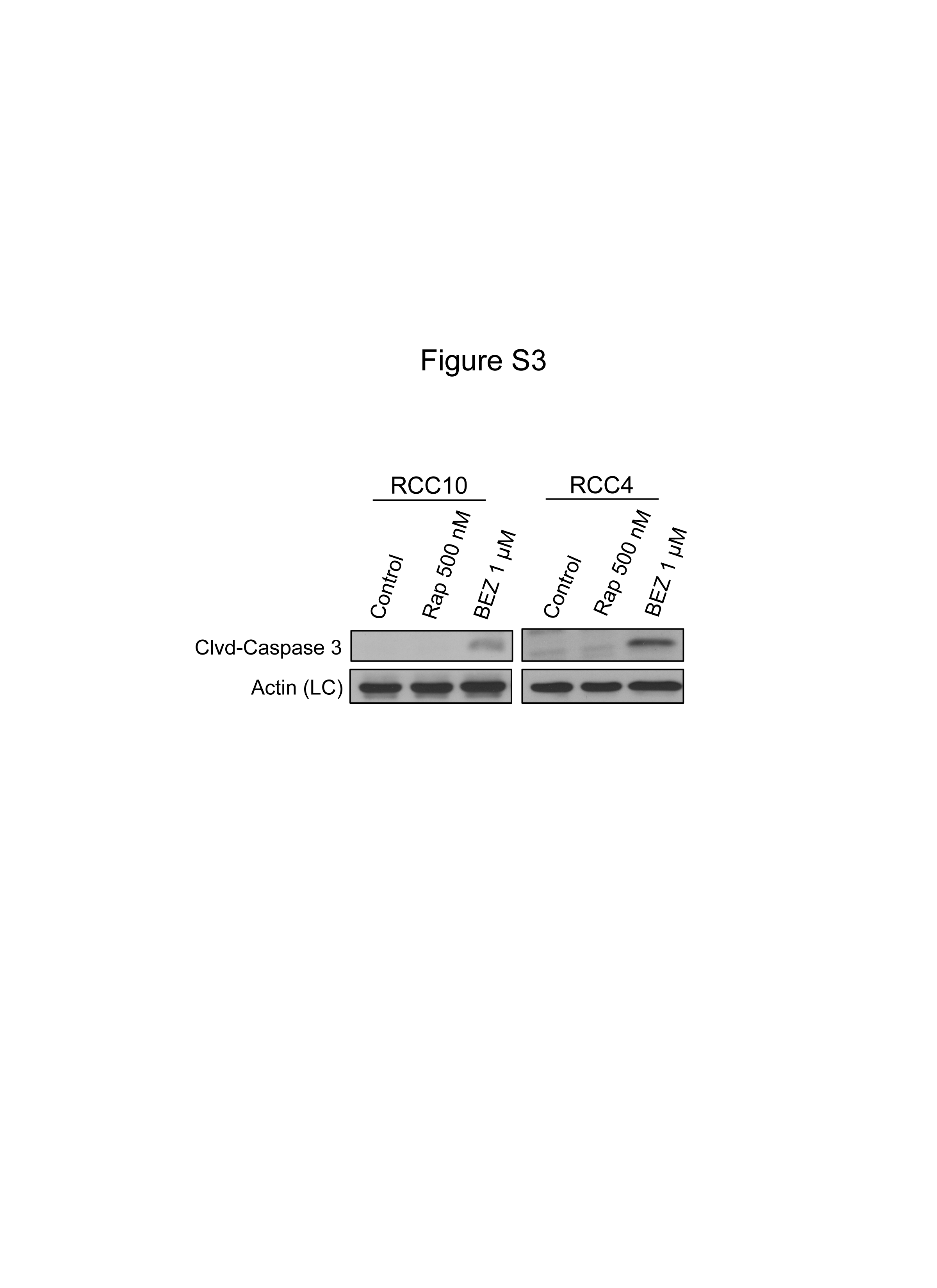

Supplement: Figure S3 — Apoptosis in response to rapamycin or BEZ235 treatment. RCC10 and RCC4 cells treated with rapamycin or BEZ235 for 24 hrs and analyzed by western blot for apoptotic marker cleaved-caspase 3. (TIF) [file pone.0104413.s003.tif]

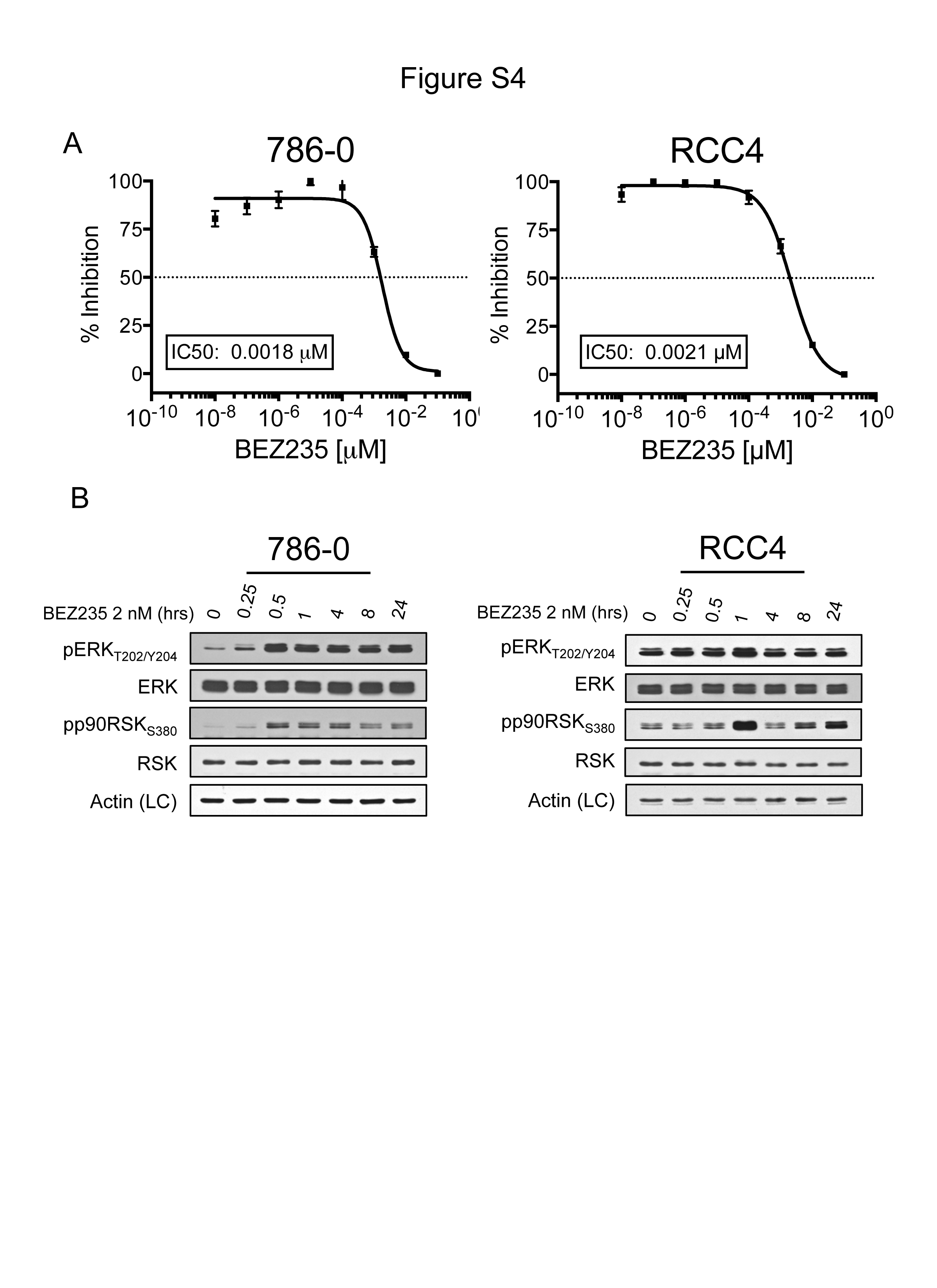

Supplement: Figure S4 — Dose titrations of BEZ235. (A) 786-0 and RCC4 cells were plated and treated with a dose titration of BEZ235 and IC50 value determined using CellTiter-Glo cell viability reagent. (B) 786-0 and RCC4 cells were treated with 2 nM BEZ235 over a 24 hr. time course and immunobloted for protein expression of mTORC1, mTORC2, and MEK/ERK signaling proteins. (TIF) [file pone.0104413.s004.tif]

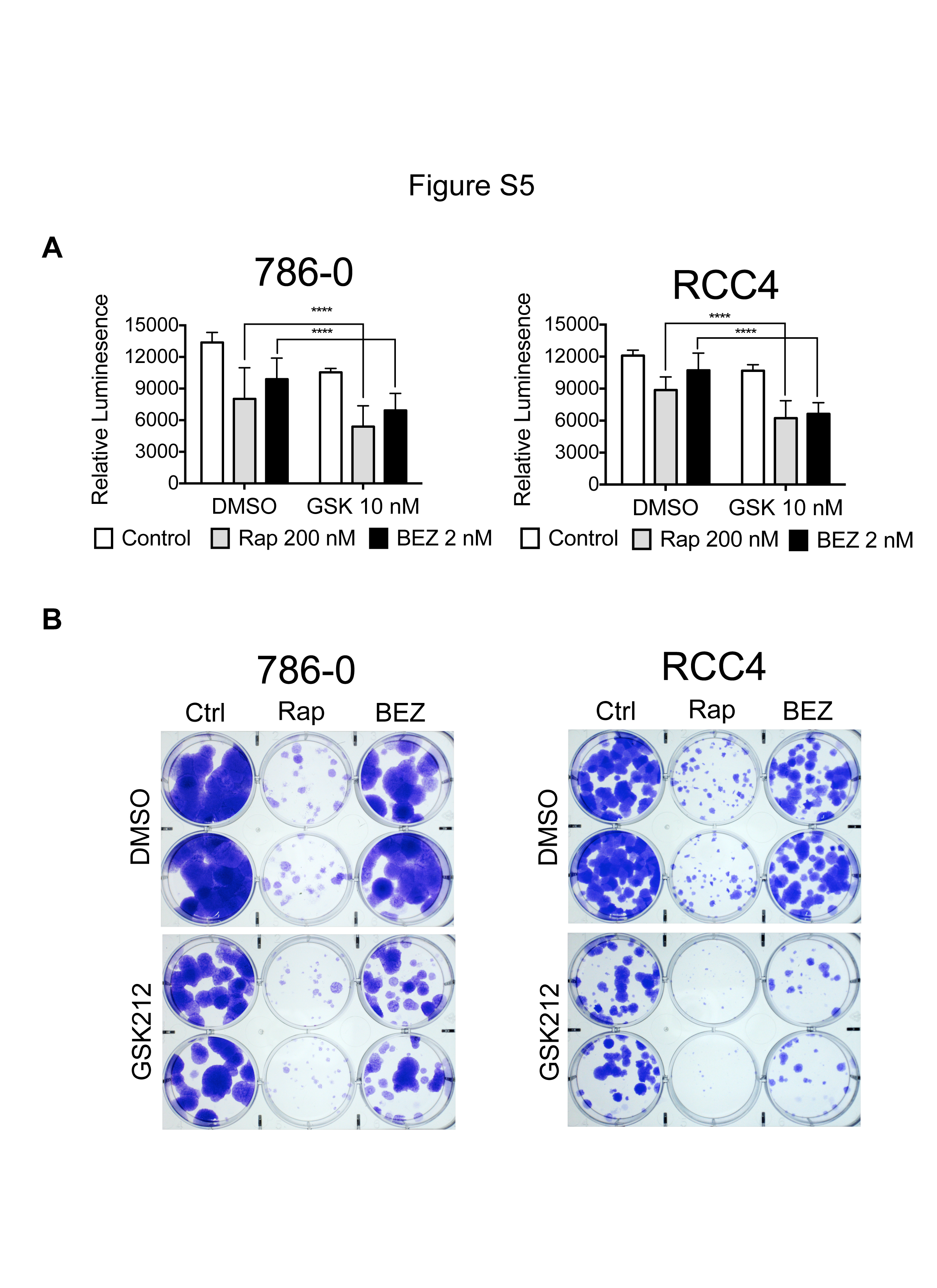

Supplement: Figure S5 — Combinatorial effects of mTOR and MEK inhibition. (A) 786-0 and RCC4 cells were treated with indicated drugs and assessed for viability on day 4 using CellTiter-Glo. Statistical significance was determined by comparing rapamycin and BEZ235 treated groups (B) 786-0 and RCC4 cells were plated, allowed to attach, and treated with 200 nM rapamycin, 2 nM BEZ235, 10 nM GSK212. Photographs of wells containing 786-0 and RCC4 cells fixed with 4% PFA and stained with 0.1% crystal violet. (TIF) [file pone.0104413.s005.tif]
