# Supplementary material for: mTOR Inhibition Induces Compensatory, Therapeutically Targetable MEK Activation in Renal Cell Carcinoma
Source: PLoS One. 2014 Sep 2;9(9):e104413. doi: 10.1371/journal.pone.0104413 (PMC4152178; doi:10.1371/journal.pone.0104413)
Supplement: Table S1 — List of antibodies used including company and catalogue number. (DOCX) [file pone.0104413.s006.docx]

**Table S1**

| **Antibody** | **Company** | **Catalogue #** |
| --- | --- | --- |
| phospho-RB Ser807/811 | CST | 9308 |
| phospho-MAPK(Erk1/2) Thr202/Tyr204 | CST | 9106 |
| phospho-AKT Thr308 | CST | 2965 |
| phospho-AKT Ser473 | CST | 4060 |
| phospho-S6 Ser235/236 | CST | 4858 |
| phospho-4E-BP1 | CST | 9459 |
| phospho-p90RSK Ser380 | CST | 9335 |
| S6 | CST | 2217 |
| 4E-BP1 | CST | 9452 |
| AKT | CST | 9272 |
| RSK1/2/3 | CST | 9355 |
| Cyclin D1 | CST | 2922 |
| Cyclin B1 | CST | 4138 |
| Raptor | CST | 2280 |
| Rictor | CST | 9476 |
| HIF-1α | CST | 3716 |
| HIF-2α | CST | 7096 |
| Cleaved-PARP | CST | 5625 |
| Caspase-3 | CST | 9662 |
| VHL | SC | sc-5575 |
| Ku80 | GT | GTX70485 |
| Tubulin | S | T5168 |
| Actin | SC | C-11 |
| MAPK(Erk1/2) | CST | 9102 |
|  |  |  |
|  |  |  |
| CST = Cell Signaling Technology |  |  |
| SC = Santa Cruz |  |  |
| AB = Abcam |  |  |
| M = Millpore |  |  |
| GT = GeneTex |  |  |
| S = Sigma |  |  |
